# Efficacy of Combinations of Methoprene and Deltamethrin as Long-Term Commodity Protectants

**DOI:** 10.3390/insects10020050

**Published:** 2019-02-05

**Authors:** Frank H. Arthur

**Affiliations:** USDA, Agricultural Research Service, Center for Grain and Animal Health Research, 1515 College Avenue, Manhattan, KS 66502, USA; frank.arthur@ars.usda.gov

**Keywords:** deltamethrin, methoprene, grains, storage, control

## Abstract

Wheat, corn, and brown rice were treated with different combinations of a deltamethrin suspension concentrate (SC) formulation and a new emulsifiable concentrate (EC) formulation, at rates of either 0.5 or 1.0 ppm alone or in combination with 1.25 and 2.5 ppm methoprene (10 treatments in all, including an untreated control). Treated commodities were stored at ambient conditions on the floor of an empty grain bin in Manhattan, KS, USA, in 5-kg lots for individual replicates. The commodities were sampled and bio-assayed every three months for 15 months by exposing 10 mixed-sex parental adults of selected adult stored product insects on 70–80 g of the commodity. For all treatments, there was no regression of declining efficacy with respect to the month. Therefore, the data were combined for analysis. On wheat and brown rice, there was no reproduction of *Rhyzopertha dominica* (Fabricius) in any of the treatments, and there was no weight loss in either commodity that was caused by feeding of the parental adults or developing progeny. There was reproduction of *Sitophilus oryzae* (L.) on wheat but, for several combinations, the EC formulation gave better suppression of progeny compared to the standard SC. However, on brown rice, only the combination of 1.0 ppm deltamethrin EC and 2.5 ppm methoprene was different than other treatments with respect to progeny development, sample weight loss caused by feeding, and weight of the feeding damage itself. Progeny production was correlated with grain damage. No progeny of *Tribolium castaneum* (Herbst) developed on the treated corn, but there was some variation in insect damage, with less damage in those treatments involving the EC formulation. Progeny production of *Sitophilus zeamais* (Motschulsky) was at the lowest in the combination of 1.0 ppm deltamethrin EC and 2.5 ppm methoprene. The resulting insect damage was the lowest in this combination as well. Results of this study were used by the registrant (Central Life Sciences) in the United States (US) to modify the commercial formulation to replace the deltamethrin SC with the EC, at label rates of either 0.5 ppm EC + 1.25 ppm methoprene, or 1.0 ppm EC + 2.5 ppm methoprene, on wheat, corn, and rice.

## 1. Introduction

When grain crops are harvested and stored, they are vulnerable to infestation from a diverse range of stored product pest insects, which are commonly referred to as either internal feeders, which complete all or most of their life cycle inside the kernel, or external feeders, which exist entirely outside of the kernel [1,2]. Examples of internal feeders include *Rhyzopertha dominica* (Fabricius), the lesser grain borer, and *Sitophilus oryzae* (L.), the rice weevil, which are common pests of stored wheat and stored rice in the United States (US) [2]. Infestations can cause economic damage through crop destruction [1,2], feeding damage that results in the loss of milling quality [3], or through rejection as commodities are transported from storage sites to mills and processing plants.

Components of insect pest management programs can include sanitation and cleaning prior to storage, aeration to modify the internal environment of the storage structure, and fumigation with phosphine to eliminate infestations that occur during storage [4]. Another viable component of management plans for stored grain management is the use of grain protectants, which are insecticides that are applied as grains and are loaded into storage [5]. In the US, there are a limited number of registered grain protectants, and not all insecticides can be applied to all grain crops [5].

In the US, wheat is harvested during the summer, with harvest times dependent on the geographic location, local conditions, and other biological and environmental factors [6]. In the Southern US, corn can be harvested and stored in late July or early August [7], and, in the south-central rice growing region of the US, the first rice crop can also be harvested and stored in late July and early August as well [8]. Thus, it could be weeks or even months before temperatures are cool enough to use aeration for bin management [6,7,8,9,10,11]. In addition, in some cases, aeration alone may not be sufficient to manage insect populations in storages located in warmer regions of the US [12,13]. Stored product insects may often be prevalent in the area surrounding grain storages, and serve as an immediate source of infestation [14,15,16].

The pyrethroid deltamethrin, trade name Centynal^®^, which is a suspension concentrate formulation (SC) was originally registered in the US at a rate of 0.5 ppm on all grain crops. It is highly effective against *R. dominica*, but it may be less effective on *S. oryzae* because longer exposure times are necessary for the control of exposed adults compared to *R. dominica* [17]. Exposed female adults of *S. oryzae* may still be able to oviposit even if they eventually succumb from exposure to deltamethrin [17]. In addition, the insecticidal formulation itself is very thick and viscous and not easy to accurately formulate in water for field applications. A new formulation of deltamethrin was developed, which is an emulsifiable concentrate (EC), that could be combined with the insect growth regulator methoprene (Diacon IGR^®^) for potentially enhanced control of *R. dominica*, *S. oryzae*, and external feeders. Methoprene will give control of R. dominica and external feeders of stored grains but does not control *Sitophilus* weevils because they oviposit inside the grain kernel and the developing larvae are not exposed to the insecticidal residues [17]. The purpose of this study was to determine relative efficacy of this new EC formulation with and without the methoprene component, at an increased application rate, for control of stored product insects on wheat, corn, and brown rice.

## 2. Materials and Methods

### 2.1. General Information

This experiment was conducted at the USDA-ARS Center for Grain and Animal Health Research (CGAHR) in Manhattan, KS, USA. There were 10 treatments: the existing SC deltamethrin (Centynal^®^, formulation used alone (label rate of 0.5 ppm) or combined with 1.25 or 2.5 ppm methoprene insect growth regulator (IGR) Diacon^®^, the new deltamethrin EC formulation used alone (at 0.5 ppm and 1.0 ppm), and each rate combined with 1.25 and 2.5 ppm methoprene). An untreated control was included as well (listed in Table 1). Both deltamethrin formulations contained 50 mg Active Ingredient (AI)/mL. These formulations were obtained from Central Life Sciences (Schaumberg, IL, USA). The methoprene formulation was 288 mg (AI)/mL and obtained from the same location. The treatment combinations are listed in Table 1. The two deltamethrin formulations were abbreviated as EC and SC, for deltamethrin while the methoprene IGR was abbreviated as M, for methoprene.

The insect species used for wheat and brown rice was adults of *R. dominica* and *S. oryzae*, while adults of *T. castaneum* and *S. zeamais* were used on corn. The *R. dominica* and *S. oryzae* used in the study for wheat were obtained from colonies reared on wheat, while the same species used on brown rice were obtained from colonies reared on brown rice. The *S. zeamais* were obtained from colonies reared on corn, but the *T. castaneum* used for corn were obtained from colonies reared on a mixture of 95% whole-wheat flour and Brewers’ yeast. All colonies were reared in Percival incubators (Perry, IA, USA) set at 27 °C and 60% relative humidity (r.h.), in continual darkness. All colonies had been in culture at the CGAHR for about 30 years.

### 2.2. Tests with Hard Red Winter Wheat

The label specifies mixing 270 mL of the deltamethrin SC or EC in 18.920 L of water to cover 27,272 kg of wheat, which is 0.7 mL of formulated spray/kg of wheat. In this study, an individual replicate consisted of 3 kg, which required 2.1 mL of spray. The rates needed for the study were 0.5 and 1.0 ppm. When either the SC or EC formulation was used for an individual replicate, to give these rates of 0.5 and 1.0 ppm either 0.35 and 0.70 mL, respectively, of the parent deltamethrin formulation that was mixed with 25 mL of distilled water. The 3 kg of wheat was spread out over a flat piece of cardboard measuring 0.6 by 0.3 m. and 2.1 mL was pipetted from the volumetric flask and applied using a Badger 100 artist’s air brush (Badger Air Brush Company, Franklin Park, IL, USA) attached to a compressor to mist the solution onto the wheat. After the wheat was treated, it was hand-mixed and then poured into an 18.9 L plastic bucket. The rates of methoprene that were needed for the test were 1.25 and 2.5 ppm or mg 1.25 and 2.5 mg AI/kg of wheat. The field volume rate if the insecticide was applied alone was also equivalent to 0.7 mL of formulated spray per kg, and the label equivalent for 1.25 and 2.5 ppm was 0.15 and 0.3 mL of the methoprene formulation, respectively, in 25 mL of water. The deltamethrin and the methoprene amounts from the respective formulation were both mixed in with 0.25 mL of water to maintain a label equivalent for volumetric application and applied at the rate of 2.1 mL of formulated spray per the 3-kg of a replicate.

The test was initiated on 15 June 2015. Each treatment was replicated four times by formulating four separate insecticide mixtures to do the application for each of the nine insecticide combinations. A set of four untreated controls of 3 kg each were sprayed with 2.1 mL of distilled water. After the wheat was treated, the 40 total buckets containing the various treatments and the replicates were placed on the floor of 27.2 metric ton (MT) capacity grain bin at the CGAHR. The next day, eight samples of about 80 g were taken from each bucket and placed in each of eight 180 mL plastic vials with a screen lid. Ten one-two-week-old mixed sex adult *R. dominica* were placed in each of four vials and ten one-two-week-old adult *S. oryzae* were placed in each of the four remaining vials. These vials, in turn, were placed in a walk-in environmental chamber set at 27 °C and 60% r.h. and held for about 10 weeks. After this time, the vials were removed from the chamber, and frozen at −18 °C to kill all adult insects. After several days, the vials were removed from the freezer and allowed to warm for at least a day on a laboratory counter under ambient conditions. The wheat in each vial was weighed. Then the adult insects were sifted off the wheat and counted. The feeding damage (frass) was collected and weighed, and the sample was weighed again to determine the amount of sample weight loss caused by the insect populations. Data for sample weight loss were converted to percentage values relative to the weight of the original sample. Subsequent bioassays were done every 3 months for 15 months.

### 2.3. Tests With Brown Rice

The brown rice was treated on 5 and 6 October with treatments 1–6 occurring on the 1st, and 7–10 occurring on the 2nd day. Again, the volumetric spray rate for rice was different from that of wheat with 1.9 mL of formulated spray per the 3 kg. The insecticides were formulated in the 25-mL volumetric flasks in accordance with label specifications for treatment on rice. There were four separate replicates for each treatment and commodity. The individual replicates were treated and then held in 19.9 L buckets on the floor of a 27.2 MT capacity empty grain bin at the CGAHR. HOBO computers with cables were placed in an untreated replicate as described for wheat, and samples were taken every 3 months and processed as described for wheat. *R. dominica* and *S. oryzae* adults were introduced into the sample vials at each bioassay time, as described for wheat.

### 2.4. Tests with Corn

The insecticides were prepared as described for wheat, following the label specifications for treatment of stored corn. The volumetric spray rate is slightly different based on the test weight of corn, so 2.0 mL of formulated spray was required for 3 kg of corn. The appropriate amounts of the formulations of deltamethrin and methoprene were mixed in 25 mL of water and applied to give the desired application rates in terms of specifications for an active ingredient. The corn was treated on 1 and 2 October, treatments 1–6 occurred on the 1st, and 7–10 occurred on the 2nd. Separate formulations were done for each of the four replicates of each of the 10 treatments. The corn replicates were treated with the airbrush as described for wheat, then placed in 18.9 L plastic buckets, and the buckets were, in turn, placed on the floor of a different empty 27.2 MT capacity grain bin at the CGAHR. Two HOBO recording computers with cables were set at 7.3 cm and at 0.30 cm below the surface in one of the untreated corn replicates. The corn was sampled as described for wheat and insects were added to the vials, except that adults of *T. castaneum* and *S. zeamais* were used for this part of the test with corn.

### 2.5. Statistical Analysis

Data were analyzed using the Statistical Analysis System (SAS, version 9.2, Cary, NC, USA). In the first analysis, data were analyzed using the Mixed Procedure to determine if there were significant differences related to months post-treatment for the four variables of analysis, sample weight loss, feeding damage, total weight loss, and progeny production. Although data varied by month and there were significant differences, there was no orderly relationship, i.e., no decline with progeny production or corresponding increase in insect damage, as residues aged (Proc Reg, *p* ≥ 0.05). Therefore, all data for months were combined for statistical analysis. Orthogonal contrasts were used to conduct pair-wise analyses of select treatments. This was done under the General Linear Models Procedure of SAS to compare the following numbered treatments in Table 1: 1 vs. 2, and 1 vs. 5 to differences between controls and the low rate of 0.5 ppm of the two deltamethrin formulations, 2 vs. 5 to compare 0.5 ppm of the SC and EC formulations, 5 vs. 6 to compare 0.5 and 1.0 ppm of the EC formulation, 7 vs. 10 to compare the 1.0 ppm of the EC formulation with and without methoprene, and 6 versus 10 to compare two EC methoprene combinations.

## 3. Results

### 3.1. Hard Red Winter Wheat

All four variables, progeny adults, sample weight loss, amount of feeding damage (frass), and total weight loss were all significant at *p* < 0.001 for *R. dominica* (*F* = 180.9, df = 9, 229, *F* = 5.5, df = 9, 229, *F* = 33.3, df = 9, 229, *F* = 12.0, df = 9, 228, respectively) under the Mixed model analysis. There was no progeny production of *R. dominica* on wheat in any of the nine treatments during the test except for 1.7 ± 0.9 and 1.6 ± 0.8 in the SC 0.5 and EC 1.0 ppm deltamethrin treatments, respectively. Frass weight averaged 6.5 ± 1.1 g in untreated controls and was <0.01 g in all treatments. Total weight loss averaged 19.6 ± 3.7% in untreated controls and <0.01% in all treatments. Female *R. dominica* lay eggs outside the kernel, and neonate larvae bore inside the kernel and then completed development to the adult stage. Thus, there is an opportunity for the neonates to be exposed to the insecticide residues before they enter the wheat kernel. There was little weight loss, frass production, or total damage in the treated wheat (Table 2).

Progeny adults, total weight loss, frass weight, and total weight loss were all significant for *S. oryzae* (*F* = 10.1, df = 9, 228, *p* < 0.001, *F* = 2.9, df = 9, 225, *p* = 0.003, *F* = 6.2, df = 9, 226, *p* < 0.001, respectively) under the mixed model analysis. In contrast to *R. dominica*, there was progeny production by *S. oryzae* on the treated wheat (Table 2), with significant differences in the orthogonal contrasts only between the deltamethrin SC and EC treatments and the controls, between the 0.5 and 1.0 EC deltamethrin treatments, and between the combination of 1.0 ppm deltamethrin EC with and without the control (Table 2). Progeny production was lowest in treatment 10, which is an indication of a possible additive effect of the deltamethrin EC plus methoprene. However, the speed of efficacy was not measured in this test, but some oviposition in the treatments apparently occurred. Only the contrast between the untreated controls and detamethrin EC at 0.5 ppm and the controls versus the 1.0 ppm plus 2.5 ppm methoprene treatment were significant for total weight loss or frass weight. Other than the possible additive effect of the highest rate of deltamethrin EC with 2.5 ppm, there was generally little improvement in efficacy gained by increasing the deltamethrin rate from 0.5 to 1.0 ppm or the inclusion of methoprene.

An analysis was done using the Correlation Procedure in SAS for both *R. dominica* and *S. oryzae* by examining all four variables including progeny production, weight loss, frass production, or total damage, over all data. All four variables were significantly positively correlated with each other (*p* < 0.01). The female *S. oryzae* inserts an egg plug into the grain kernel, and the developing larva is not normally exposed to a residual insecticide. Therefore, methoprene is not generally effective on weevils because of the limited larval exposure. Since deltamethrin is a contact insecticide for all insect life stages, it was expected that there would be some efficacy against adult weevils, but, for complete control, adult females should be killed before they can oviposit.

### 3.2. Brown Rice

There was no progeny production or insect damage resulting from the exposure of parental adult *R. dominica* on treated brown rice (Table 3). While progeny production of *S. oryzae* was generally lower in treatments than in untreated controls, there was considerable variation among the nine treatments. Progeny production of *S. oryzae* appeared to be much greater on brown rice than wheat. Although some values for each of the four analysis variables (progeny production, sample wt. loss, frass weight, and total damage) appear to be numerically different for the analysis variables, the Bonferroni test is conservative and did not show significant differences between treatments. Brown rice is rice with the protective husk removed. Therefore, it is easier for the female weevil to penetrate compared to wheat or rough rice with the protective husk. Previous studies show that *R. dominica* did not develop well on rough rice and may require a crack or split in the hull or husk to gain entry [18,19,20], which is why we used brown rice for this current study. All four variables were significantly and positively correlated with each other (*p* < 0.01) for both *R. dominica* and S. *zeamais* on brown rice.

### 3.3. Corn

Progeny production of *T. castaneum* was low on untreated controls possibly because this species is not a primary feeder on stored corn, but, nevertheless, no progeny was produced on the treated corn. Hence, contrasts were not done. There was some variation in insect damage, particularly when the high rate of methoprene was used. This could be attributable to extension of the larval stage, which can occur when larvae of *T. castaneum* are exposed to IGRs. The treatments involving the EC formulation of deltamethrin, alone or in combination with methoprene, were significantly different from the SC formulation for the two damage variables. Overall, there was little damage in any of the treatments with the deltamethrin EC (Table 4). Progeny production of *S. zeamais* on corn was variable in all treatments, but the combination of 1.0 ppm deltamethrin EC and 2.5 ppm methoprene gave the lowest progeny and the least amount of weight loss and feeding damage (Table 5).

## 4. Discussion

Previous studies indicate that neonate *R. dominica* larvae are very susceptible to methoprene applied to different stored grains, even though the exposure time could be very brief before the neonates bore into grain kernels [21]. Although there is no indication of time of death of larvae that were able to bore into a grain kernel, the reduced adult emergence indicates that the neonate absorbs enough of the methoprene so that the exposed larva cannot emerge from the kernel as an adult. *R. dominica* was also susceptible to the SC formulation of deltamethrin, alone or in combination with methoprene, and there was no apparent loss of efficacy with the new EC formulation. In addition, the new EC formulation was much easier to formulate in water compared to the SC formulation, which made it easier to apply the treatments involving the new EC formulation to the commodity.

There was some evidence that the EC formulation of deltamethrin was more effective than the SC formulation for control of *S. oryzae* on wheat but was less effective on brown rice. This was possibly due to a potential capacity for greater progeny production on brown rice when compared to wheat. Other studies have also shown reduced effectiveness when stored product insects are exposed for short time intervals on grains treated with deltamethrin or exposed on packaging that have deltamethrin incorporated into the laminate layers of the packaging [22,23,24].

All treatments were effective against *T. castaneum* for 15 months on corn, which was expected since this species is an external feeder and all life stages would be exposed to the insecticide residues. Data also indicate that the new EC formulation may give some level of control of *S. zeamais* on corn even though additional tests may be necessary due to the low progeny production in untreated controls in the current study. Given the concerns regarding resistance to the fumigant phosphine in the US [25,26,27], there is a need for increased use of grain protectants in the management programs for stored bulk grains. Protectants such as those evaluated in this study could be combined with other management strategies, including aeration to cool and modify the storage bin temperature. Use of a grain protectant does not preclude the use of phosphine in case fumigation becomes necessary to eliminate an infestation that arises during storage.

In summary, the EC formulation of deltamethrin gave increased efficacy over the current SC formulation for residual control of stored product insects. The registrant of the insecticide could use this data to improve the combination pyrethroid-methoprene combination by substituting the new formulation. It may be beneficial to provide several application rates for the product, depending on primary target species for the protectant application.

## 5. Conclusions

The website states that a conclusion is optional. As a result of reviewer comments, I deleted the Conclusion and moved the paragraph to the Discussion section. The paper was accepted without a conclusion section.

## Figures and Tables

**Table 1:** List of insecticide treatments and abbreviations used in the following tables.

1. UTC, untreated control
2. Suspension Concentrate (SC) formulation of deltamethrin applied at 0.5 ppm
3. SC applied at 0.5 ppm + 1.25 ppm methoprene (M)
4. SC at 0.5 ppm + 2.5 ppm M
5. EC formulation of deltamethrin applied at 0.5 ppm
6. EC applied at 1.0 ppm
7. EC applied at 0.5 ppm + 1.25 ppm M
8. EC applied at 0.5 ppm + 2.5 ppm M
9. EC applied at 1.0 ppm + 1.25 ppm M
10. EC applied at 1.0 ppm + 2.5 ppm M

**Table 2:** Progeny production, total weight loss (WL in %), and feeding damage (Frass wt. in g) (means ± SE) from exposure of 10 mixed-sex parental adult *S. oryzae* on untreated wheat and wheat treated with the insecticide combinations in Table 1. *F* and *P* values given for selected orthogonal contrasts.

	Treatment	#	Mean ± SE	Contrast	*F*	*p*
Progeny	Untreated	1	154.4 ± 16.5			
	SC0.5	2	52.8 ± 15.1	1 vs. 2	30.9	<0.001
	SC0.5 + M1.25	3	57.6 ± 12.2	1 vs. 5	11.4	<0.001
	SC0.5 + M2.5	4	36.6 ± 12.2	2 vs. 5	4.4	0.036
	EC0.5	5	91.4 ± 16.9	5 vs. 6	12.7	<0.001
	EC1.0	6	26.0 ± 8.2	3 vs. 7	0.7	0.419
	EC0.5 + M1.25	7	42.7 ± 13.8	4 vs. 8	0.7	0.396
	EC0.5 + M2.5	8	52.2 ± 13.5	7 vs. 9	4.5	0.034
	EC1.0 + M1.25	9	30.1 ± 12.4	6 vs. 10	1.5	0.228
	EC1.0 + M2.5	10	3.7 ± 2.8	1 vs. 10	65.8	<0.001
Total %WL	Untreated	1	2.23 ± 0.40			
	SC0.5	2	1.12 ± 0.38	1 vs. 2	5.2	0.023
	SC0.5 + M1.25	3	1.08 ± 0.33	1 vs. 5	2.9	0.090
	SC0.5 + M2.5	4	0.80 ± 0.37	2 vs. 5	0.3	0.559
	EC0.5	5	1.40 ± 0.35	5 vs. 6	4.8	0.030
	EC1.0	6	0.37 ± 0.19	3 vs. 7	0.0	0.967
	EC0.5 + M1.25	7	1.06 ± 0.33	4 vs. 8	0.4	0.525
	EC0.5 + M2.5	8	1.09 ± 0.49	7 vs. 9	3.3	0.071
	EC1.0 + M1.25	9	0.58 ± 0.28	6 vs. 10	0.1	0.740
	EC1.0 + M2.5	10	0.20 ± 0.05	1 vs. 10	17.9	<0.001
Frass wt.	Untreated	1	0.46 ± 0.07			
	SC0.5	2	0.16 ± 0.06	1 vs. 2	17.2	<0.001
	SC0.5 + M1.25	3	0.18 ± 0.04	1 vs. 5	6.1	0.041
	SC0.5 + M2.5	4	0.10 ± 0.04	2 vs. 5	2.7	0.100
	EC0.5	5	0.28 ± 0.06	5 vs. 6	8.8	0.003
	EC1.0	6	0.07 ± 0.02	3 vs. 7	0.0	0.871
	EC0.5 + M1.25	7	0.17 ± 0.06	4 vs. 8	0.7	0.381
	EC0.5 + M2.5	8	0.17 ± 0.06	7 vs. 9	4.8	0.029
	EC1.0 + M1.25	9	0.10 ± 0.05	6 vs. 10	0.6	0.452
	EC1.0 + M2.5	10	0.09 ± 0.05	1 vs. 10	39.1	<0.001

**Table 3:** Progeny production, total weight loss (WL in %), and feeding damage (Frass wt. in g) (means ± SE) from exposure of 10 mixed-sex parental adult *S. oryzae* on untreated brown rice and brown rice treated with the insecticide combinations Table 1. *F* and *p* values given for selected orthogonal contrasts.

	Treatment	#	Mean ± SE	Contrast	*F*	*p*
Progeny	Untreated	1	154.8 ± 23.9			
	SC0.5	2	53.3 ± 6.3	1 vs. 2	36.1	<0.001
	SC0.5 + M1.25	3	58.5 ± 7.8	1 vs. 5	16.9	<0.001
	SC0.5 + M2.5	4	77.9 ± 10.8	2 vs. 5	3.6	0.085
	EC0.5	5	85.4 ± 12.5	5 vs. 6	1.7	0.199
	EC1.0	6	63.7 ± 11.3	3 vs. 7	3.3	0.071
	EC0.5 + M1.25	7	89.2 ± 9.2	4 vs. 8	0.1	0.741
	EC0.5 + M2.5	8	83.5 ± 10.3	7 vs. 9	5.1	0.024
	EC1.0 + M1.25	9	73.4 ± 11.4	6 vs. 10	0.6	0.448
	EC1.0 + M2.5	10	50.8 ± 5.8	1 vs. 10	37.9	<0.001
Total WL	Untreated	1	5.10 ± 1.06			
	SC0.5	2	2.94 ± 1.10	1 vs. 2	2.5	0.115
	SC0.5 + M1.25	3	3.06 ± 0.84	1 vs. 5	6.1	0.014
	SC0.5 + M2.5	4	3.01 ± 0.92	2 vs. 5	0.8	0.371
	EC0.5	5	1.70 ± 1.60	5 vs. 6	0.5	0.489
	EC1.0	6	2.67 ± 0.70	3 vs. 7	0.3	0.599
	EC0.5 + M1.25	7	2.32 ± 0.49	4 vs. 8	0.1	0.888
	EC0.5 + M2.5	8	2.82 ± 0.71	7 vs. 9	0.1	0.792
	EC1.0 + M1.25	9	3.58 ± 1.71	6 vs. 10	0.3	0.601
	EC1.0 + M2.5	10	1.96 ± 0.43	1 vs. 10	5.3	0.023
Frass wt.	Untreated	1	0.76 ± 0.18			
	SC0.5	2	0.34 ± 0.10	1 vs. 2	6.3	0.013
	SC0.5 + M1.25	3	0.31 ± 0.09	1 vs. 5	3.9	0.050
	SC0.5 + M2.5	4	0.44 ± 0.12	2 vs. 5	0.3	0.588
	EC0.5	5	0.43 ± 0.13	5 vs. 6	0.5	0.467
	EC1.0	6	0.30 ± 0.11	3 vs. 7	0.3	0.611
	EC0.5 + M1.25	7	0.40 ± 0.07	4 vs. 8	0.1	0.791
	EC0.5 + M2.5	8	0.49 ± 0.12	7 vs. 9	1.0	0.314
	EC1.0 + M1.25	9	0.49 ± 0.17	6 vs. 10	0.2	0.643
	EC1.0 + M2.5	10	0.22 ± 0.04	1 vs. 10	10.1	0.002

**Table 4:** Total weight loss (WL in %, means ± SE) from exposure of 10 mixed-sex parental adult *T. castaneum* on corn treated with the insecticide combinations Table 1. *F* and *p* values given for selected orthogonal contrasts. Progeny production in untreated wheat was 13.4 ± 2.4 and frass weight was 0.41 ± 0.04 g. No progeny production or frass in the treatments.

	Treatment	#	Mean ± SE	Contrast	*F*	*p*
Progeny	Untreated	1	13.4 ± 2.4			
Total WL	Untreated	1	1.49 ± 0.16			
	SC0.5	2	0.34 ± 0.13	1 vs. 2	55.5	<0.001
	SC0.5 + M1.25	3	0.35 ± 0.13	1 vs. 5	62.8	<0.001
	SC0.5 + M2.5	4	0.66 ± 0.29	2 vs. 5	0.2	0.633
	EC0.5	5	0.26 ± 0.07	5 vs. 6	0.1	0.151
	EC1.0	6	0.21 ± 0.06	3 vs. 7	1.0	0.267
	EC0.5 + M1.25	7	0.19 ± 0.04	4 vs. 8	0.0	0.274
	EC0.5 + M2.5	8	0.37 ± 0.18	7 vs. 9	1.9	0.975
	EC1.0 + M1.25	9	0.26 ± 0.06	6 vs. 10	1.7	0.421
	EC1.0 + M2.5	10	0.41 ± 0.15	1 vs. 10	48.8	<0.001

**Table 5:** Progeny production, total weight loss (WL in %), and feeding damage (Frass wt. in mg) (means ± SE) from exposure of 10 mixed-sex parental adult *S. zeamais* on untreated corn and corn treated with the insecticide combinations Table 1. *F* and *p* values given for selected orthogonal contrasts.

	Treatment	#	Mean ± SE	Contrast	*F*	*p*
Progeny	Untreated	1	26.4 ± 5.9			
	SC0.5	2	10.8 ± 5.1	1 vs. 2	8.5	0.004
	SC0.5 + M1.25	3	7.3 ± 4.1	1 vs. 5	6.0	0.156
	SC0.5 + M2.5	4	10.8 ± 4.4	2 vs. 5	0.2	0.633
	EC0.5	5	13.4 ± 5.0	5 vs. 6	2.1	0.151
	EC1.0	6	5.7 ± 3.0	3 vs. 7	1.2	0.267
	EC0.5 + M1.25	7	1.4 ± 1.0	4 vs. 8	1.2	0.274
	EC0.5 + M2.5	8	5.0 ± 2.6	7 vs. 9	0.0	0.975
	EC1.0 + M1.25	9	2.0 ± 1.7	6 vs. 10	0.7	0.403
	EC1.0 + M2.5	10	1.0 ± 1.1	1 vs. 10	22.3	<0.001
Total WL	Untreated	1	1.12 ± 0.16			
	SC0.5	2	0.84 ± 0.26	1 vs. 2	0.8	0.377
	SC0.5 + M1.25	3	0.72 ± 0.26	1 vs. 5	0.8	0.386
	SC0.5 + M2.5	4	0.99 ± 0.24	2 vs. 5	0.0	0.987
	EC0.5	5	0.85 ± 0.23	5 vs. 6	0.5	0.479
	EC1.0	6	0.63 ± 0.24	3 vs. 7	0.8	0.369
	EC0.5 + M1.25	7	0.43 ± 0.20	4 vs. 8	0.5	0.465
	EC0.5 + M2.5	8	0.76 ± 0.30	7 vs. 9	0.1	0.792
	EC1.0 + M1.25	9	0.39 ± 0.09	6 vs. 10	0.2	0.716
	EC1.0 + M2.5	10	0.52 ± 0.13	1 vs. 10	3.8	0.054
Frass wt.	Untreated	1	0.25 ± 0.11			
	SC0.5	2	0.14 ± 0.07	1 vs.2	2.7	0.104
	SC0.5 + M1.25	3	0.08 ± 0.04	1 vs. 5	2.3	0.132
	SC0.5 + M2.5	4	0.19 ± 0.07	2 vs. 5	0.0	0.904
	EC0.5	5	0.15 ± 0.04	5 vs. 6	0.7	0.994
	EC1.0	6	0.09 ± 0.04	3 vs. 7	0.0	0.316
	EC0.5 + M1.25	7	0.08 ± 0.05	4 vs. 8	1.0	0.922
	EC0.5 + M2.5	8	0.12 ± 0.04	7 vs. 9	0.0	0.805
	EC1.0 + M1.25	9	0.05 ± 0.02	6 vs. 10	0.6	0.363
	EC1.0 + M2.5	10	0.07 ± 0.04	1 vs. 10	6.7	0.012
